# A bibliometric analysis: Current status and frontier trends of Schwann cells in neurosciences

**DOI:** 10.3389/fnmol.2022.1087550

**Published:** 2023-01-12

**Authors:** Yan Wang, Shiwen Zhang, Jincao Zhi, Meiling Huang, Fei Pei

**Affiliations:** ^1^Rehabilitation Center of the Second Affiliated Hospital, Heilongjiang University of Chinese Medicine, Harbin, China; ^2^Graduate School of Heilongjiang University of Chinese Medicine, Harbin, China

**Keywords:** Schwann cells, neurosciences, CiteSpace, VOSviewer, bibliometric analysis

## Abstract

**Background:**

This review aims to present a comprehensive bibliometric analysis related to Schwann cells (SCs) in neurosciences from 2012 to 2021.

**Methods:**

We used the Web of Science core collection database to obtain publications on SCs in the field of neurosciences from 2012 to 2021. The obtained data were further visually analyzed by using CiteSpace, VOSviewer, and an online bibliometric platform.

**Results:**

We retrieved a total of 1,923 publications related to SCs in neurosciences. The number of publications in this field fluctuates steadily each year, and the number of citations is increasing year by year. The United States is leading the field, with LERU and the University OF London as influential institutions, Jessen KR and Feltri ML as the most representative authors, and GLIA and JOURNAL OF NEUROSCIENCE as authoritative journals in the field. Meanwhile, we predict that a more in-depth study of autophagy and phagocytosis functions of SCs and the key regulator c-Jun will probably be a hot spot for future research.

**Conclusion:**

This study summarizes the current research results and predicts research trends for further research, which will facilitate researchers in quickly understanding the current state of research in the field while referring to new research directions.

## 1. Introduction

Schwann cells (SCs), which originate from the neural crest, are the most abundant glial cells in the peripheral nervous system (PNS) with both myelinated and non-myelinated types (Harty and Monk, 2017; Zhang et al., 2020; Saiki et al., 2021). Myelin SCs wrap around the axons of motor and sensory neurons to form myelin sheaths. Non-myelinated SCs, Remak cells, wrap around small diameter axons of 0.5–1.5 μm to form Remak bundles (Feltri et al., 2016; Sapkota and Dougherty, 2020). These cells ensure normal development of the PNS and play an essential role in regeneration after injury (Maugeri et al., 2020). After peripheral nerve injury, myelinated and non-myelinated SCs are reprogrammed with specialized repair promoting phenotypes, called repair SCs, which can provide biochemical signals and spatial cues to promote survival and axonal regeneration of damaged neurons. Importantly, SCs have become an important subject of intensive research in neurosciences due to their key role in nerve injury repair (Xia et al., 2020).

In contrast to systematic and scoping reviews, bibliometric analysis allows for quantitative analysis of published literature in specific scientific fields through the use of mathematical and statistical methods, which will help us to better understand the structure of knowledge and significant advances in certain areas of research. To date, bibliometric analysis has been widely used in public health and clinical researches (Patil et al., 2020; Wilson et al., 2021; Zhang et al., 2022). However, the bibliometric analysis of SCs in neurosciences is very scarce, and to our knowledge, only in Zhang et al. (2012) based on Web of Science conducted a brief bibliometric analysis of the annual publication output, distribution by journal, distribution by the institution and top-cited articles of SCs from 2002 to 2011. Therefore, a comprehensive high-quality bibliometric analysis, especially from 2012 to the present, is necessary to quickly understand the hot research topics and explore the frontier trends related to SCs in the field of neurosciences in recent years. In this study, we conducted a comprehensive bibliometric analysis of SCs research in neurosciences from 2012 to 2021, focusing on the annual number of publications and cited times, countries, funds, institutions, authors, journals, references, and keywords. Our study aims to summarize and sort out the mainstream research themes and, more importantly, highlight emerging themes to help researchers with new ideas for future research.

## 2. Materials and methods

### 2.1. Data collection

**Figure 1:**
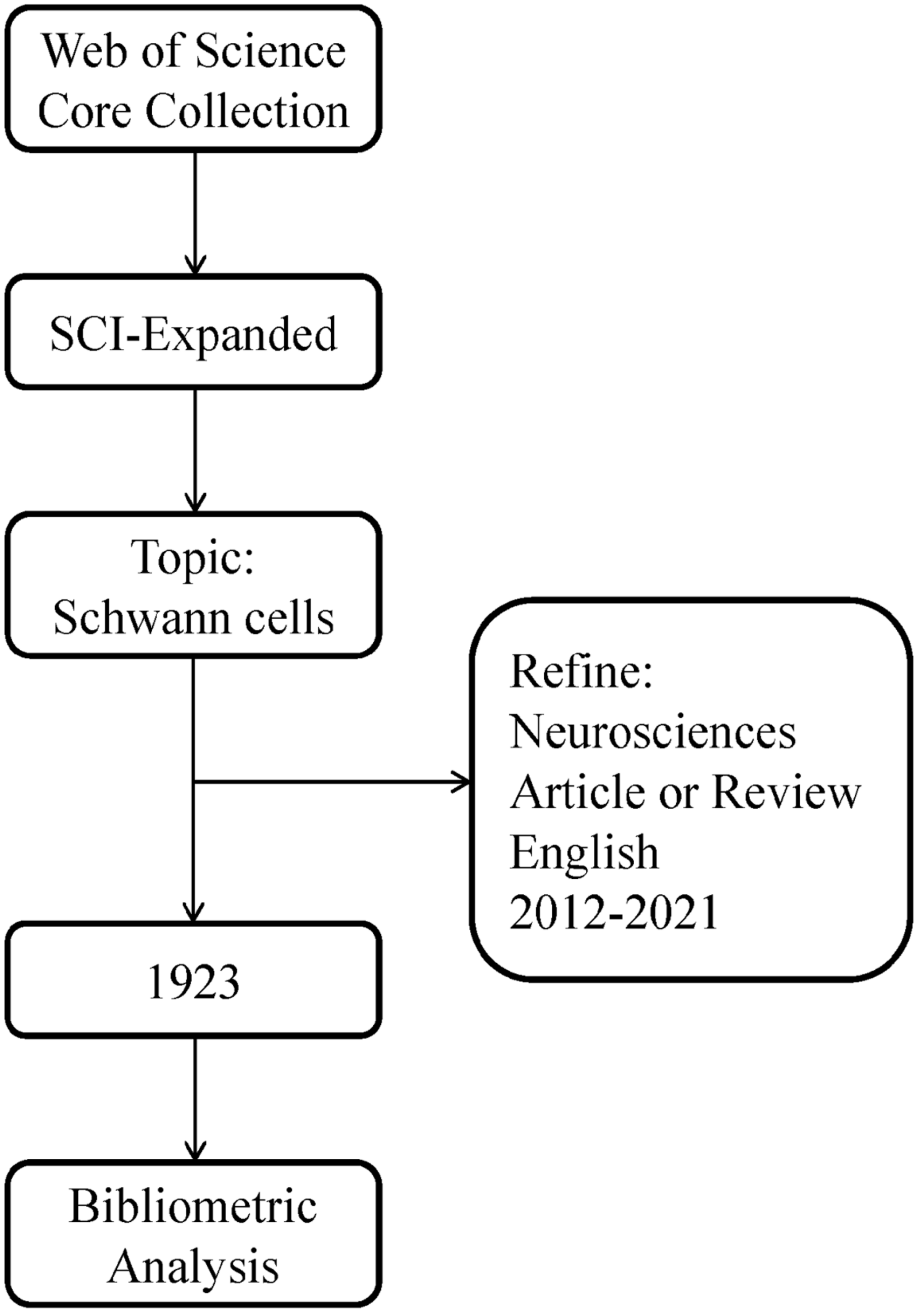
Flowchart steps of the search strategy.

We conducted a comprehensive online search of the literature related to SCs using the Web of Science Core Collection database^1^, and the search strategy is shown in Figure 1. The index, category, document type, language type, and time span of the search were refined and a total of 1,923 documents were retrieved, which were exported as “full record and cited references” or UTF-8 for further analysis.

### 2.2. Data analysis

We extracted the annual number of articles issued, citations, H-index, and the average number of citations per item (ACI) from the analysis of the results, and obtained the impact factor (IF) and quartile categories from the 2020 Journal Citation Report of the Web of Science database. The UTF-8 format data were imported into the bibliometric online analysis platform^2^ for collaborative relationship analysis between countries/regions. For co-authorship as well as co-citation, co-occurrence, cluster, and burst analysis, we applied CiteSpace^3^ (Version 5.8.R3) and VOSviewer^4^ (Version 1.6.17) software to visualize bibliometric data, which were imported in “full record and cited reference” format. CiteSpace developed by Chen, as one of the most commonly used visual analysis software in bibliometrics, is used to observe research hotspots and trends in a specific field and visually present them in the form of a graph (Synnestvedt et al., 2005). VOSviewer, developed by Prof. van Eck and Waltman in 2009, can visualize scientific landscapes *via* network, coverage, or density maps (van Eck and Waltman, 2010).

## 3. Results

### 3.1. Publication and cited times

**Figure 2:**
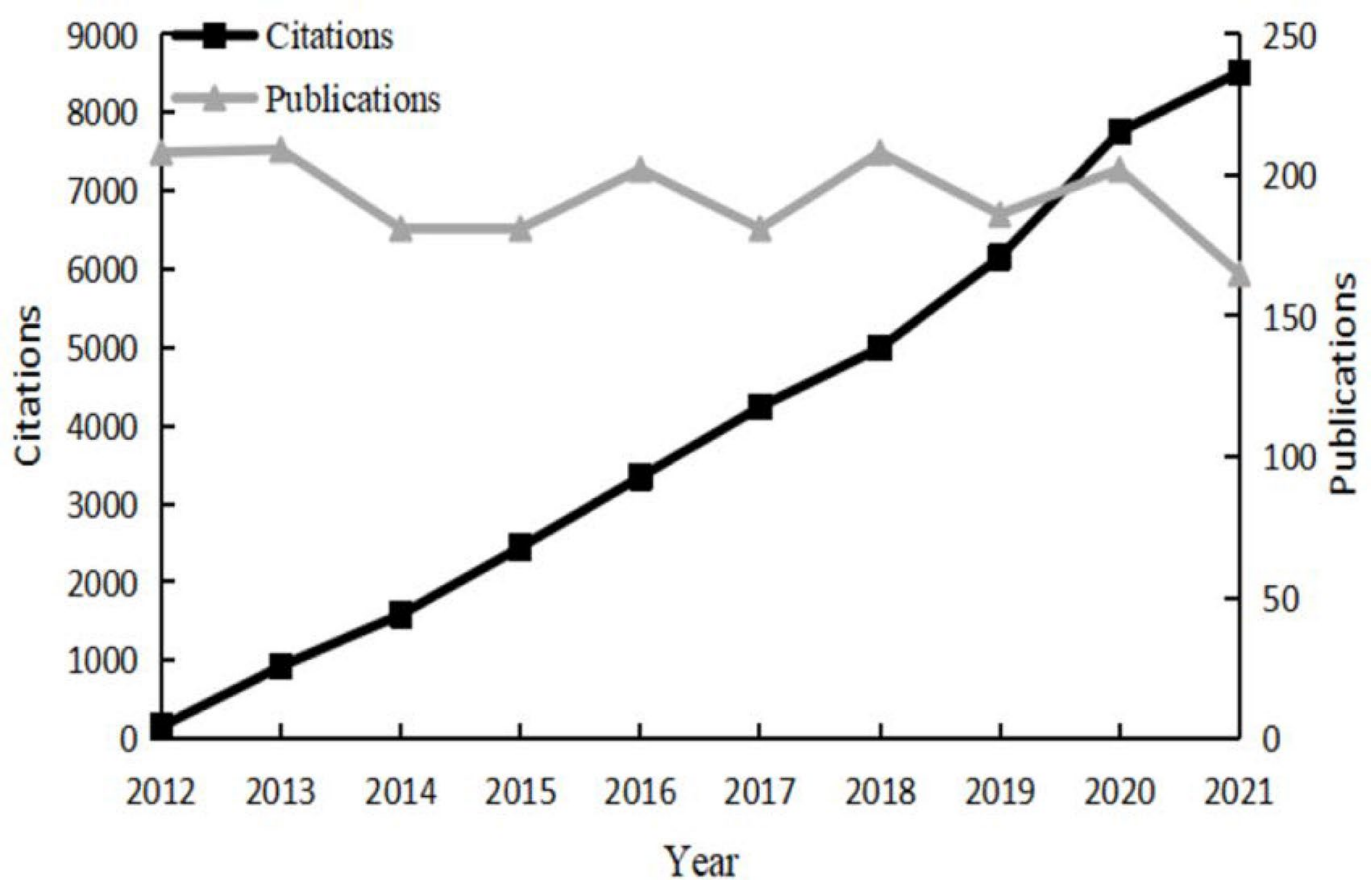
The annual publications and citations.

After filtering by the above search process, 1,923 papers were included, including 1,548 articles and 375 reviews. The annual number of publications and citations from 2012 to 2021 is clearly shown in Figure 2. We can discover that from 2012 to 2020, the annual number of publications shows a small fluctuation, and the number of publications in 2021 decreases more. From the perspective of citations, the total citations were 42,625, and the remaining 36,542 after removing self-citations. From 2012 to 2021, the annual citations increased year by year, with the highest in 2021, reaching 8,512.

### 3.2. Countries and funds

**Figure 3:**
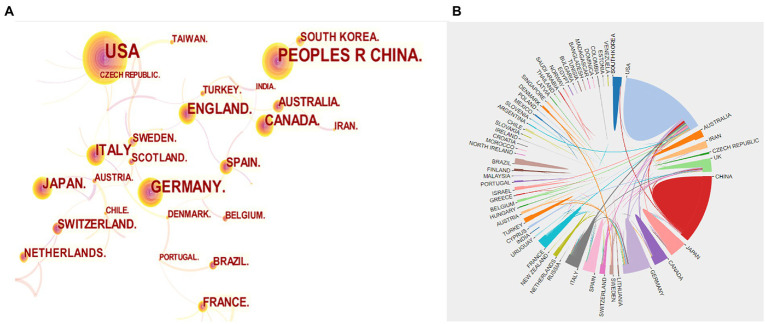
**(A)** Visualization map of countries co-occurrence. **(B)** Collaboration analysis among different countries.

In Figure 3A, the number of papers published by different countries was shown, with the larger circles indicating more publications. We can observe from this that the United States (651), China (445), and Germany (227) are the top three countries with the highest number of publications. The cooperation between different countries/regions was presented in Figure 3B. Since the thicker the line between two countries means more cooperation between them, we can see that the United States cooperates most with other countries, and among them, Germany and China have a thicker line with the United States, which proves that the United States communicates more closely with them. In addition, according to Table 1, we conducted an analysis of funding agencies and found that the top three most prolific funding agencies were, the National Institutes Of Health (459), the United States Department Of Health Human Services (459), and the National Institute Of Neurological Disorders Stroke Ninds (311), notably, they are all from the United States, which emphasizes the influence of the United States in the field.

**Table 1:** Top 5 prolific funders.

Rank	Funders	Publications	% of 1,923
1	National Institutes of Health	459	23.87
2	United States Department of Health Human Services	459	23.87
3	National Institute of Neurological Disorders Stroke Ninds	311	16.17
4	National Natural Science Foundation of China	289	15.03
5	European Commission	193	10.04

### 3.3. Institutions

**Figure 4:**
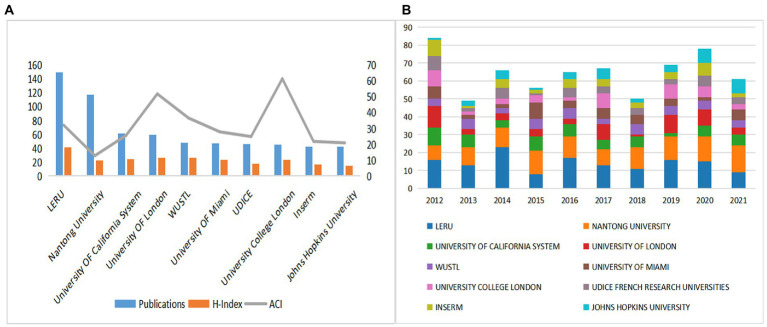
**(A)** The publications, H-indexes, and ACIs of the top 10 most prolific institutions. **(B)**The publications of the top 10 most prolific institutions each year.

The publication counts, H-indexes, and ACIs of the top 10 most prolific institutions were displayed in Figure 4A. The top 5 institutions in terms of publication counts were League of European Research Universities (LERU) (150), Nantong University (117), the University of California system (61), and the University of London (59). H-index can be used to reflect the quantity and level of academic outputs (Miao et al., 2022). LERU ranked first in the H Index (41), followed by the University of London (26) and Washington University in St. Louis (WUSTL) in the United States (26). ACI is another tool for assessing the value of a paper (Wu et al., 2021). University College London (61.44), University of London (51.88), and WUSTL (36.6) were the top three institutions with the highest ACI, and LERU ranked fourth at 32.13. We can recognize from this that LERU and the University of London have stronger integrated competencies and have shown their influence in the field. It is worth thinking that Nantong University had a low H-index and ACI ranking despite the high number of publications. We suggested that Nantong University should strengthen cooperation with other countries and improve the quality of articles to increase its influence in this field. In addition, we quantified in Figure 4B the number of papers published per year by the top 10 prolific institutions. LERU had the highest number of publications in 2014 with a more stable overall fluctuation in publications per year, and the University of London had the highest number of 12 in 2012, but the number of publications started to decline in the following years, and importantly, in the last 5 years, both institutions showed a significant increase in publications in 2019, indicating SCs were more popular in 2019.

### 3.4. Authors

**Figure 5:**
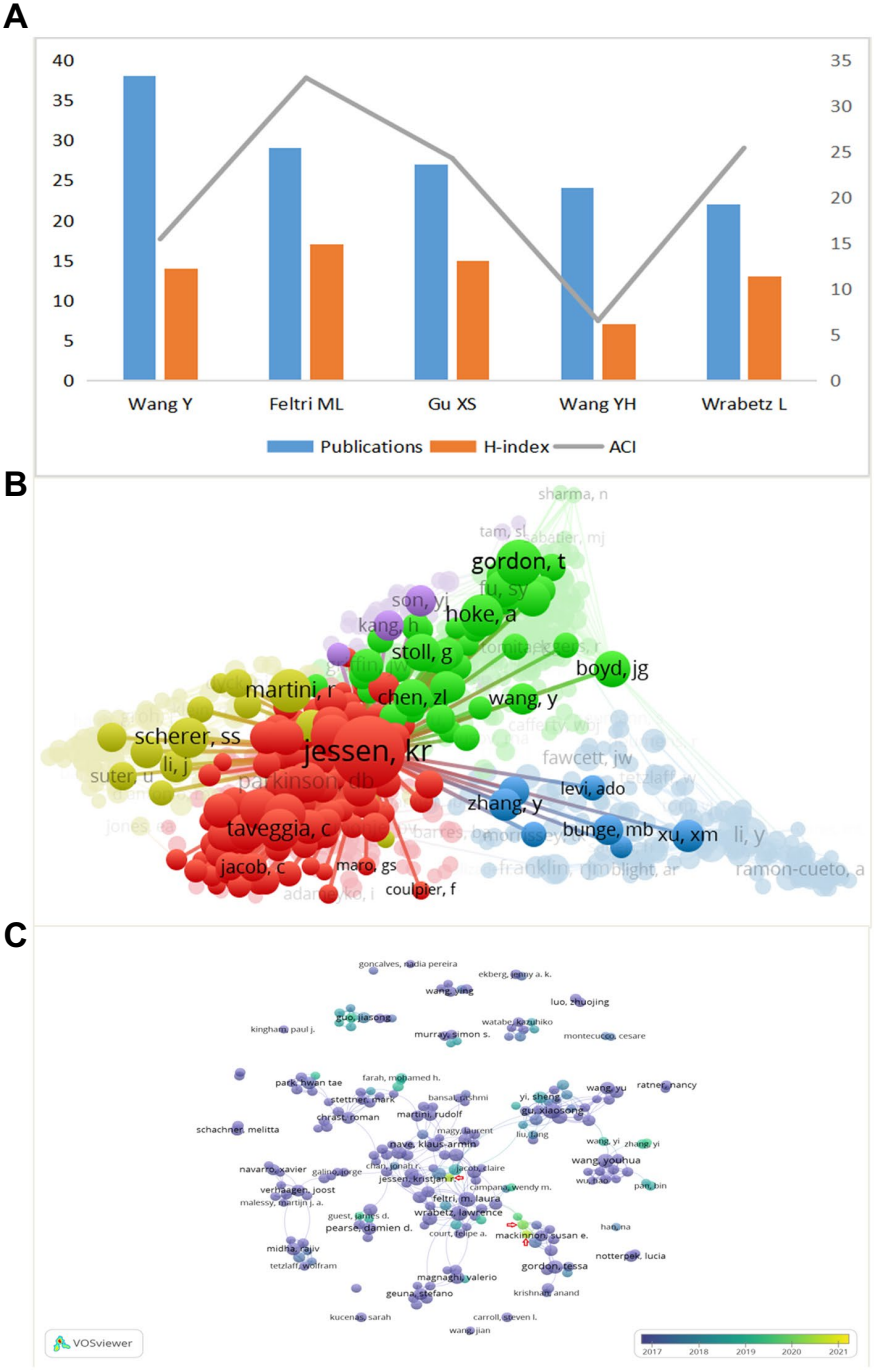
**(A)** The publications, H-index, and ACI of the top 5 most prolific authors. **(B)** Network visualization map of authors co-citation analysis. **(C)** Coverage visualization map of authors co-authorship analysis.

As shown in Figure 5A, the highest number of publications was Wang Y (38), followed by Feltri ML (29) and Gu XS (27). Feltri ML had the highest H-index of 17, followed by Gu XS and Wang Y. Also, the top three ACI were Feltri ML, Wrabetz L and Gu XS. We can conclude that Feltri ML was leading in terms of the number of publications, H-index, and ACI, which proved that he is an author of high authority in the field. His articles represent hot topics in a part of the field. For example, Feltri ML et al. recently found that CC2D1B, a member of the Lgd/CC2D1 protein family, plays a role in developmental myelination in the central nervous system and suggested that CC2D1B may be involved in gene regulation during myelination in optic oligodendrocytes (Acheta et al., 2022). The citation frequency is another important indicator to measure the influence, as Figure 5B shows, the larger the circle means the more citations. Jessen KR was cited much more frequently than other authors, ranked first, and cooperated closely with other authors, which also represented the great influence of Jessen KR in the field. Furthermore, in the coverage visualization map in Figure 5C, nodes are marked by different colors according to the average year of appearance, with authors appearing relatively early in the field closer to purple, and nodes marked in yellow are likely to represent authors with younger research in the field. We can observe that the author Min Qing, as well as Pan Deng, Snyder-Warwick, Alison k are new players in this field in recent years.

### 3.5. Journals

Papers related to SCs in neurosciences were published in 162 journals from 2012 to 2021. We listed the top ten journals in this field based on the number of publications, as demonstrated in Table 2. NEURAL REGENERATION RESEARCH was in the first place with 176 published articles, GLIA was in the second place with 163 articles, followed by JOURNAL OF NEUROSCIENCE with 124 articles. Besides, the JIF of a journal is another important parameter to evaluate the value of the journal itself and the publications included in it. Among the top 10 academic journals, GLIA had the highest JIF at 7.452, followed by JOURNAL OF NEUROSCIENCE at 6.167, and both were classified as Q1. Also, the top three cited journals were JOURNAL OF NEUROSCIENCE (4688), GLIA (3767), and EXPERIMENTAL NEUROLOGY (2821). Based on the above, we believe that GLIA and JOURNAL OF NEUROSCIENCE are the more authoritative journals in this field.

**Table 2:** Top 10 most productive journals.

Rank	Journal	Count	IF (2020)	Quartile in category (2020)	Citation
1	NEURAL REGENERATION RESEARCH	176	5.135	Q2	2,384
2	GLIA	163	7.452	Q1	3,767
3	JOURNAL OF NEUROSCIENCE	124	6.167	Q1	4,688
4	EXPERIMENTAL NEUROLOGY	85	5.33	Q2	2,821
5	FRONTIERS IN CELLULAR NEUROSCIENCE	70	5.505	Q1	1,318
6	NEUROSCIENCE LETTERS	66	3.046	Q3	1,088
7	NEUROSCIENCE	50	3.59	Q3	984
8	MOLECULAR NEUROBIOLOGY	47	5.59	Q1	1,052
9	MUSCLE NERVE	38	3.217	Q3	495
10	FRONTIERS IN MOLECULAR NEUROSCIENCE	37	5.639	Q1	699

### 3.6. Citations

**Figure 6:**
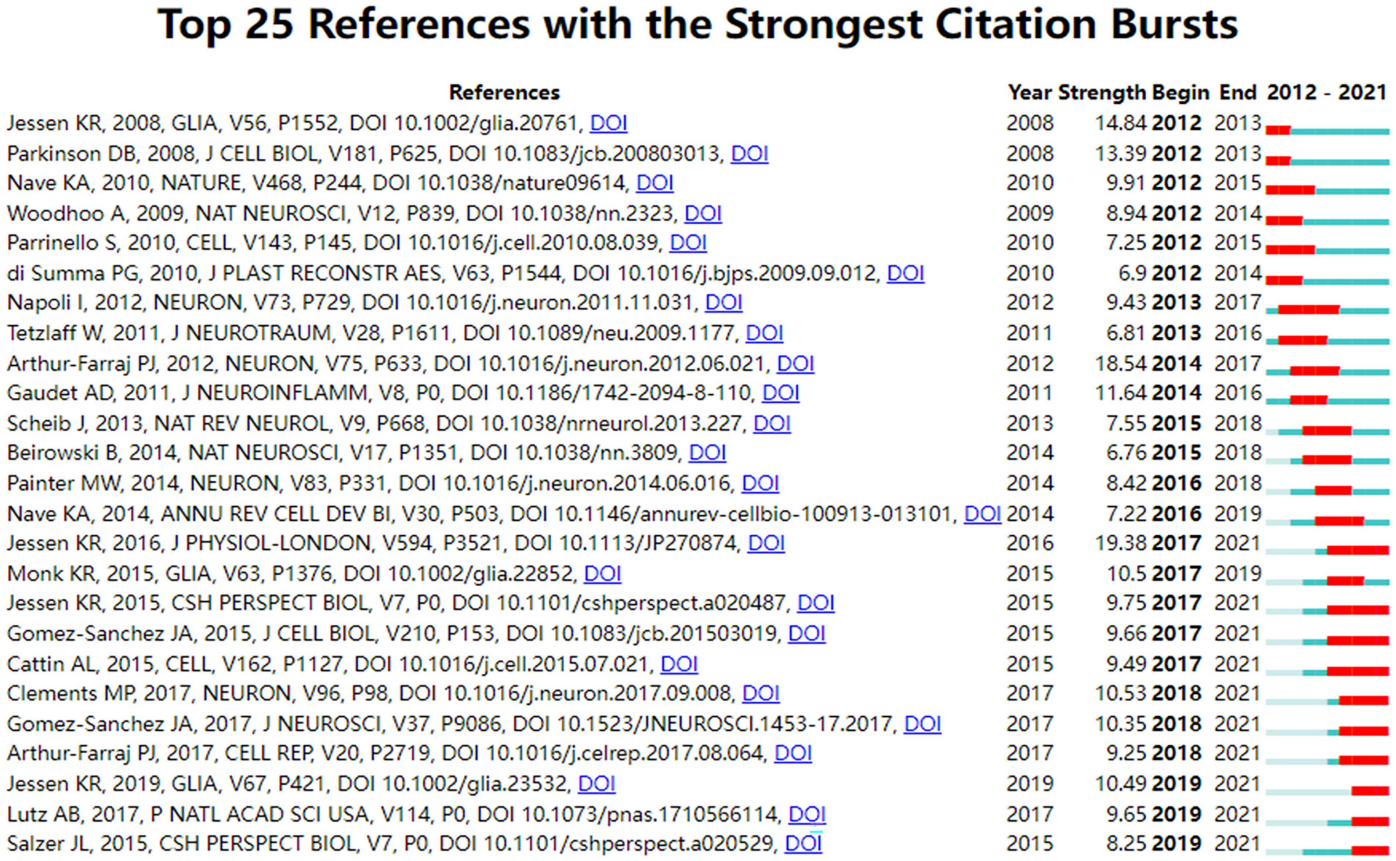
Top 25 references with the strongest citation bursts.

Citation analysis is an important indicator in bibliometric studies. Table 3 showed the top 10 most cited literatures, including 5 mechanistic studies (Gaudet et al., 2011; Jessen et al., 2015; Monk et al., 2015; Salzer, 2015; Jessen and Mirsky, 2016) and 5 experimental studies (Arthur-Farraj et al., 2012; Napoli et al., 2012; Stassart et al., 2013; Cattin et al., 2015; Gomez-Sanchez et al., 2015), which focused on the development and function of SCs. Interestingly, the top four most-cited papers were all written or directed by Jessen KR, who works at the University of London, reflecting Jessen KR’s great influence in the field. Citation bursts refer to references that caught the attention of scholars in a specific field at a specific time interval, and whose analysis can be used to observe the evolution of a field of knowledge and to predict frontier trends. In Figure 6, the timeline was shown in blue and the interval at the time of the burst was shown in red, indicating the start year, the end year, and the duration of the burst. Of these burst citations, the shortest burst duration for SCs-related publications in neurosciences was 1 year and the longest was 4 years. Notably, the 40% citation burst ended in 2021 or later, which focused on new advances in SCs in myelin clearance and remyelination (Cattin et al., 2015; Gomez-Sanchez et al., 2015; Jessen et al., 2015; Salzer, 2015; Brosius Lutz et al., 2017; Clements et al., 2017; Gomez-Sanchez et al., 2017) as well as transcription factors, epigenetic mechanisms and signaling cascades that regulate the repair SCs (Jessen and Mirsky, 2016; Arthur-Farraj et al., 2017; Jessen and Arthur-Farraj, 2019), suggesting that these research topics have been receiving attention in recent years and are expected to be a focus for researchers in the future.

**Table 3:** Top 10 most cited references.

Rank	Title	Author	Year	Citation
1	The repair Schwann cell and its function in regenerating nerves	Jessen KR	2016	111
2	C-Jun Reprograms Schwann Cells of Injured Nerves to Generate a Repair Cell Essential for Regeneration	Arthur-Farraj PJ	2012	87
3	Schwann Cells: Development and Role in Nerve Repair	Jessen KR	2015	59
4	Schwann cell autophagy, myelinophagy, initiates myelin clearance from injured nerves	Gomez-Sanchez JA	2015	54
5	A Central Role for the ERK-Signaling Pathway in Controlling Schwann Cell Plasticity and Peripheral Nerve Regeneration *In Vivo*	Napoli I	2012	52
6	New Insights on Schwann Cell Development	Monk KR	2015	50
7	A role for Schwann cell-derived neuregulin-1 in remyelination	Stassart RM	2013	48
8	Wallerian degeneration: Gaining perspective on inflammatory events after peripheral nerve injury	Gaudet AD	2011	44
9	Macrophage-Induced Blood Vessels Guide Schwann Cell-Mediated Regeneration of Peripheral Nerves	Cattin AL	2015	44
10	Schwann Cell Myelination	Salzer JL	2015	42

### 3.7. Keywords

**Figure 7:**
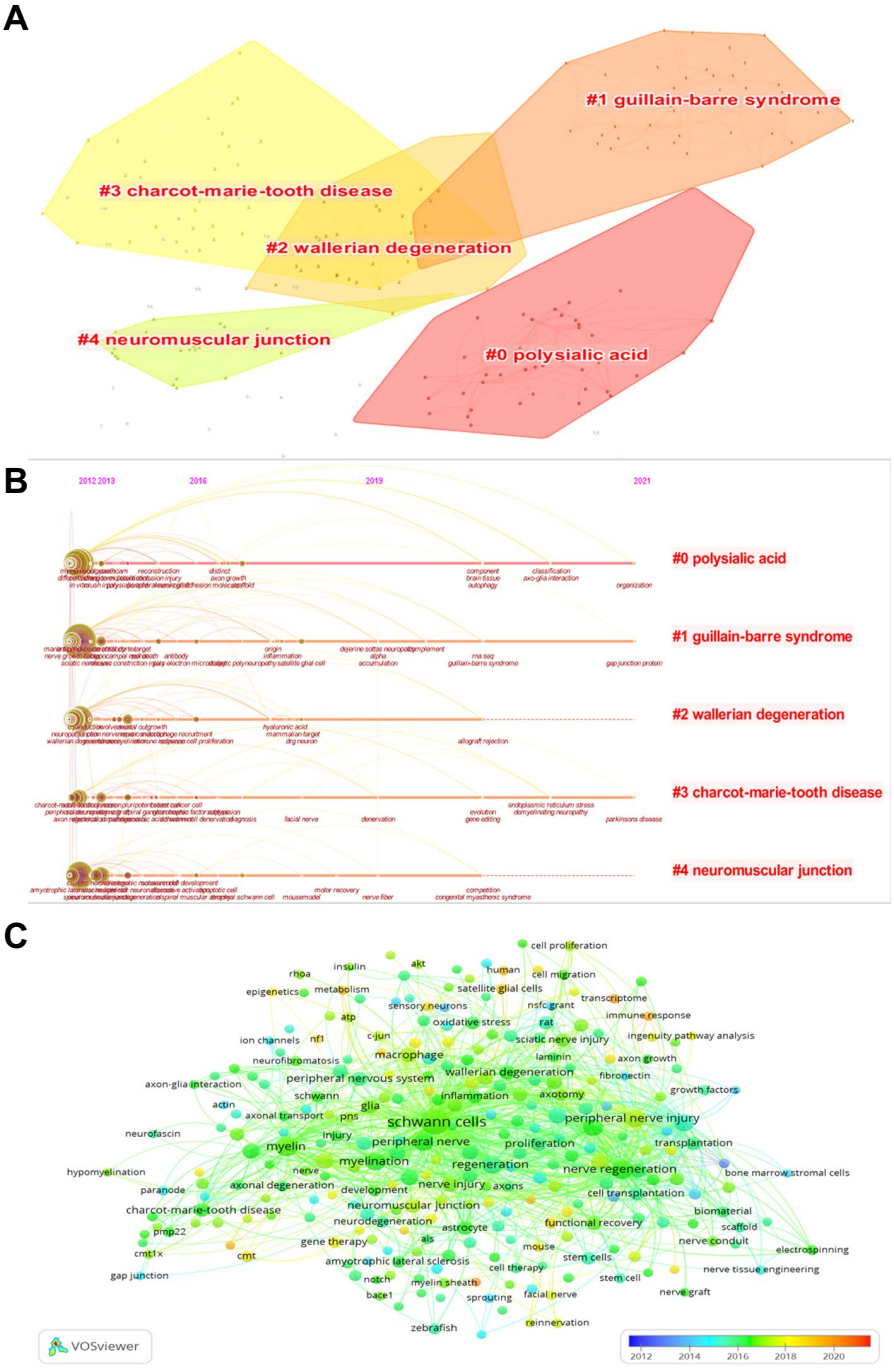
**(A)** Visualization map of keywords cluster analysis. **(B)** Visualization map of the timeline view. **(C)** Coverage visualization map of keywords co-occurrence analysis.

The modularity value (*Q* value) and the average silhouette value (*S* value) are two important parameters to evaluate the importance of cluster structure, and when *Q* > 0.3 and *S* > 0.7, it indicates that the clusters are significant. In this study, it can be evidenced from Figure 7A that the *Q* value was 0.8235 and the mean *S* value was 0.9702, indicating that these clusters are effective. The top five clusters were displayed in the figure which were, “polysialic acid” (#0), “Guillain-barre syndrome (GBS)” (#1), “Wallerian degeneration” (#2), “Charcot–Marie–tooth disease (CMT)” (#3), and “neuromuscular junction” (#4). For further study, the timeline view was shown in Figure 7B, with the bold timeline indicating that the clustering topic was a hot spot during this period. In particular, polysialic acid, GBS, and CMT were hot until 2021 and will probably last until today. GBS and CMT as demyelinating diseases are greatly associated with SCs, and SCs are being investigated as therapeutic agents for demyelinating diseases, and further sorting out the function of SCs will help in the development of treatment (Bhatheja and Field, 2006; Moss et al., 2021). We again performed the analysis of keyword co-occurrence by using VOSviewer’s coverage visualization map. As shown, trigeminal nerve, transcriptome, and immune response are the emerging hot keywords in recent years in Figure 7C. Reported that the immune response plays an important role in the early stages after nerve injury, transcriptome analysis is now often used to further investigate the molecular mechanisms of the immune response after peripheral nerve injuries to provide a scientific basis for more effective treatment of peripheral nerve injuries (He et al., 2021).

## 4. Discussion

A more in-depth keyword and citation analysis will help us to understand the current research priorities and trends in a particular field. Therefore, we identified the research hotspots and frontiers of schwann cells related research in the field of neuroscience, as described below. SCs are the major glial cells in the PNS and play a key role in the development, maintenance, and regeneration of peripheral nerves (Gomez-Sanchez et al., 2022). SCs possess an autocrine survival circuit. After nerve injuries, these SCs lost contact with axons, are transformed into cells that specifically support regeneration, which is a key step in the regenerative role of repair SCs in the PNS (Gomez-Sanchez et al., 2017; Sardella-Silva et al., 2021). The distal nerve of the injured site occurs disintegration after nerve injuries, called Wallerian degeneration, which was first studied by Waller (1851). Wallerian degeneration often occurs between 36 h and 7–14 days after injury. Myelin degradation occurs after axon degeneration, and the mechanism of the role of SCs in it is not fully understood to date (Huang et al., 2022). Since degraded myelin was found in autophagic vesicles in cells after injury, Gomez-Sanchez et al. found that Schwann cell autophagy was the main mechanism for myelin clearance after nerve transection injury (Gomez-Sanchez et al., 2015). Subsequently, Lutz et al. validated this conclusion in a crush model and demonstrated that autophagy alone was not sufficient to exhibit the full myelin clearance of SCs after nerve crush injury. Subsequently, it was found that Schwann cell phagocytosis, mediated by the TAM (Axl, Mer) receptor and *via* macrophage assist, could increase the possibility that SCs could reach their full myelin clearance potential (Brosius Lutz et al., 2017). The autophagy and phagocytosis of SCs are critical for the clearance of myelin, axons, and cellular debris after injury. Remarkably, when myelin is excessively degraded or myelin clearance is abnormal, nerve regeneration and remyelination will be traumatized, which in turn may lead to the development of various neural diseases (Martini et al., 2013). Gomez-Sanchez et al. observed evidence of enhanced autophagic activation in the uninjured state of the nerve in a mouse model of the most common inherited demyelinating neurological disease in humans (Gomez-Sanchez et al., 2015). The study to determine the cellular and molecular mechanisms behind the autophagy and phagocytosis of SCs is of great importance to future researchers, and the identification of this mechanism may provide new ideas for the treatment of demyelinating diseases.

After myelin debris has been removed, SCs and macrophages co-secrete trophic factors and other cytokines to promote axonal repair and regeneration (Balakrishnan et al., 2021). Macrophages secrete VEGF-A to promote vascularization to relieve hypoxia at the site of injury and facilitate the migration of SCs (Cattin et al., 2015). The repair SCs are 7–10 times longer than immature SCs, and the increased length ensures the formation of continuous regenerative trajectory Bungner bands formed by overlapping SCs, allowing for better extension and reconnection of regenerated axons along these tubes (Gomez-Sanchez et al., 2017). However, the repair cell phenotype is unstable, which may be related to the prolonged lack of axonal contact in distal SCs during human neural regeneration (Jessen and Mirsky, 2016). The regulation and maintenance of the repair cell phenotype have been a hot topic of interest in recent years. The most specific example is c-Jun, proven to be a key regulator for reprogramming of SCs to repair post-damage cells and maintain the repair phenotype (Arthur-Farraj et al., 2017; Jessen and Arthur-Farraj, 2019; Jessen and Mirsky, 2019). In addition, a 28-fold elevation of c-Jun in SCs was found to lead to hypomyelination pathology in c-Jun-pure overexpressing mice, suggesting that c-Jun may be a potential target for demyelinating neurological diseases and deserves to be investigated in depth by other researchers (Jessen and Mirsky, 2021). Demyelinating diseases, such as GBS and CMT, are a group of diseases that pose a significant burden on the global economy and society. In general, the prognosis of these diseases is poor and there is no effective and reliable cure. In recent decades, several studies have revealed the neuroprotective role of SCs in the PNS (Palomo Irigoyen et al., 2018). And through the above studies, we believe that a deeper understanding of the molecular mechanisms behind the autophagy and phagocytosis of SCs and the key regulator of repair SCs, c-Jun, will help to better understand these diseases and may lead to new therapeutic approaches.

## 5. Limitation

To our knowledge, the present study is the first attempt to conduct a comprehensive bibliometric analysis of papers related to SCs in neurosciences from 2012 to 2021. Although this paper has made some meaningful findings, there are some limitations at the same time. Due to the formatting requirements of the CiteSpace software, all data were retrieved and downloaded from the Web of Science database, excluding other medical databases such as PubMed and Scopus, and we have restricted the indexing, article type, and language type of the search, which may result in the omission of some high-quality articles. However, it is undeniable that we believe that the volume of data retrieved is large enough and can adequately reflect the current state of research.

## 6. Conclusion

We searched and analyzed 1,923 English publications in neurosciences related to SCs published from 2012 to 2021. Our findings show that the number of annual publications in this study fluctuates more steadily, with a greater decline in 2021 and an increase in the number of citations year by year, with a high number of 8,512 citations in 2021. The United States was leading the field, with LERU and the University OF London as influential institutions, Jessen KR and Feltri ML as the most influential authors in the field, and GLIA and JOURNAL OF NEUROSCIENCE as authoritative journals in the field. We predict that a deeper understanding of autophagy and phagocytosis functions of SCs and the regulatory factor c-Jun may be a hot spot for future research. In conclusion, this study summarized the data from published research papers and provided a reference for further research related to SCs in the field of neurosciences.

## Data availability statement

The original contributions presented in the study are included in the article/supplementary material, further inquiries can be directed to the corresponding author.

## Author contributions

YW and SZ: conception, design, drafting the article, and study supervision. SZ, JZ, MH, and FP: acquisition of data. YW, SZ, JZ, MH, and FP: statistical analysis. All authors contributed to the article and approved the submitted version.

## Funding

This work was supported by the National Natural Science Foundation of China (82274623 and 81973926) and the Natural Science Foundation of Heilongjiang Province (LH2021H090).

## Conflict of interest

The authors declare that the research was conducted in the absence of any commercial or financial relationships that could be construed as a potential conflict of interest.

## Publisher’s note

All claims expressed in this article are solely those of the authors and do not necessarily represent those of their affiliated organizations, or those of the publisher, the editors and the reviewers. Any product that may be evaluated in this article, or claim that may be made by its manufacturer, is not guaranteed or endorsed by the publisher.

## Footnotes

^1^ https://wcs.webofknowledge.com

^2^ http://bibliometric.com

^3^ https://citespace.podia.com

^4^ http://www.vosviewer.com
